# Selective Lentiviral Gene Delivery to CD133-Expressing Human Glioblastoma Stem Cells

**DOI:** 10.1371/journal.pone.0116114

**Published:** 2014-12-26

**Authors:** N. Sumru Bayin, Aram S. Modrek, August Dietrich, Jonathan Lebowitz, Tobias Abel, Hae-Ri Song, Markus Schober, David Zagzag, Christian J. Buchholz, Moses V. Chao, Dimitris G. Placantonakis

**Affiliations:** 1 Department of Neurosurgery, NYU School of Medicine, New York, NY, United States of America; 2 Helen L. and Martin S. Kimmel Center for Stem Cell Biology, NYU School of Medicine, New York, NY, United States of America; 3 Medical Scientist Training Program, NYU School of Medicine, New York, NY, United States of America; 4 Molecular Biotechnology and Gene Therapy, Paul-Ehrlich-Institut, Langen, Germany; 5 Department of Cell Biology, NYU School of Medicine, New York, NY, United States of America; 6 Ronald O. Perelman Department of Dermatology, NYU School of Medicine, New York, NY, United States of America; 7 Department of Pathology, NYU School of Medicine, New York, NY, United States of America; 8 German Cancer Consortium, Heidelberg, Germany; 9 Skirball Institute, NYU School of Medicine, New York, NY, United States of America; 10 Brain Tumor Center, NYU School of Medicine, New York, NY, United States of America; University of Alabama at Birmingham, United States of America

## Abstract

Glioblastoma multiforme (GBM) is a deadly primary brain malignancy. Glioblastoma stem cells (GSC), which have the ability to self-renew and differentiate into tumor lineages, are believed to cause tumor recurrence due to their resistance to current therapies. A subset of GSCs is marked by cell surface expression of CD133, a glycosylated pentaspan transmembrane protein. The study of CD133-expressing GSCs has been limited by the relative paucity of genetic tools that specifically target them. Here, we present CD133-LV, a lentiviral vector presenting a single chain antibody against CD133 on its envelope, as a vehicle for the selective transduction of CD133-expressing GSCs. We show that CD133-LV selectively transduces CD133+ human GSCs in dose-dependent manner and that transduced cells maintain their stem-like properties. The transduction efficiency of CD133-LV is reduced by an antibody that recognizes the same epitope on CD133 as the viral envelope and by shRNA-mediated knockdown of CD133. Conversely, the rate of transduction by CD133-LV is augmented by overexpression of CD133 in primary human GBM cultures. CD133-LV selectively transduces CD133-expressing cells in intracranial human GBM xenografts in NOD.SCID mice, but spares normal mouse brain tissue, neurons derived from human embryonic stem cells and primary human astrocytes. Our findings indicate that CD133-LV represents a novel tool for the selective genetic manipulation of CD133-expressing GSCs, and can be used to answer important questions about how these cells contribute to tumor biology and therapy resistance.

## Introduction

Glioblastoma multiforme (GBM) is a deadly primary brain malignancy, with 10,000 new cases in the US annually (http://www.cbtrus.org). Despite aggressive surgery and concomitant chemo and radiotherapy, median survival is only 14.6 months [1]. Stem-like cells within these tumors, namely Glioblastoma Stem Cells (GSCs), have the ability to self-renew, differentiate into tumor lineages and initiate tumors in immunodeficient animal models [2], [3], [4], [5], [6]. More importantly, they are believed to be the reason for tumor recurrence by overcoming current therapies via cell-intrinsic and tumor microenvironment-dependent mechanisms [7], [8], [9], [10], [11]. Therefore, they represent crucial therapeutic targets.

**Figure 1:**
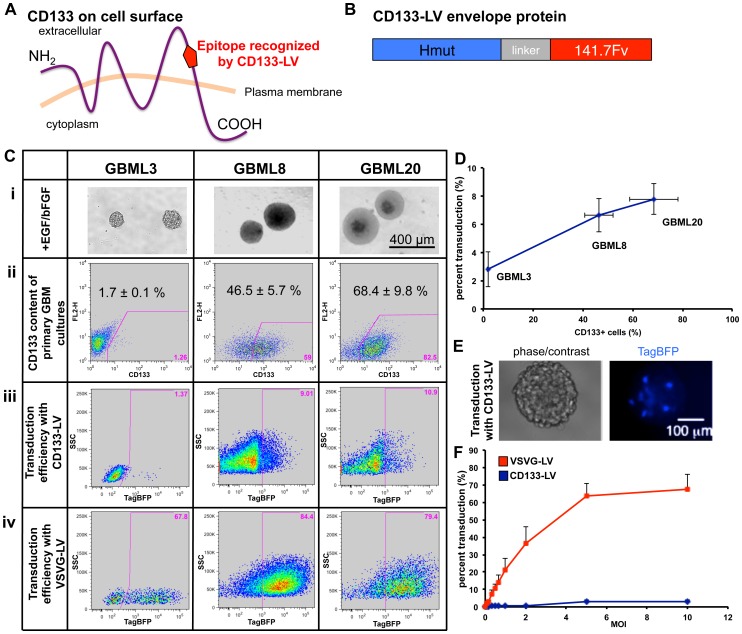
CD133-LV transduces CD133+ cells in primary human GBM cultures *in vitro*. **A**. Plasma membrane topology of CD133. CD133-LV recognizes an epitope on the second extracellular loop of the glycoprotein. **B**. Envelope protein of CD133-LV (Hmut: mutant hemagglutinin, 141.7Fv: single chain antibody against 293C3 epitope on second extracellular loop of CD133). Hmut and 141.7Fv are separated by a linker peptide. **C**. Observations collected from 3 primary GBM cultures. **i**. Primary GBM cultures are maintained as tumorspheres in suspension using media supplemented with EGF and bFGF. **ii**. Cell surface CD133 expression varies among different GBM lines, as assayed by flow cytometry. **iii,iv**. Flow cytometric analysis of transduction efficiency with CD133-LV (**iii**) and VSVG-LV (**iv**) expressing TagBFP. **D**. CD133-LV transduction efficiency correlates with the CD133 content of three primary GBM cultures (MOI = 5). **E**. Fluorescent microscopy shows a human GBM tumorsphere with scattered cells transduced with CD133-LV and thereby expressing TagBFP. This image was taken three days after incubation of cells with CD133-LV. **F**. Transduction of GBML3 cultures (CD133 content is 1.7±0.1%) with CD133-LV is dose-dependent and transduction efficiency is significantly lower than with pantropic VSVG-LV.

CD133 (PROM1) is a pentaspan transmembrane glycoprotein found on the plasma membrane (**Fig. 1A**). Its mouse homolog was identified in neuroepithelial stem cells, while the human homolog was discovered in human hematopoietic stem cells [12], [13], [14], [15]. CD133 cell surface expression has been linked to stem cells, including endothelial progenitor cells, hematopoietic stem cells, fetal brain stem cells, embryonic epithelium, prostate epithelial stem cells, myogenic cells, and ependymal cells in the adult brain; as well as cancer stem cells in leukemia, teratocarcinoma, medulloblastoma, retinoblastoma and GBM, among other tumors [16], [17], [18], [19], [20], [21], [22], [23], [24]. Within GBM, CD133+ tumor cells initiate tumors in animal models more efficiently than their CD133- counterparts, supporting the hypothesis that they represent stem-like cancer cells [3].

Despite its extensive use as a marker to isolate stem cells, CD133's function, its role in cellular signaling and its significance in GBM biology are not well characterized. Global targeted deletion of CD133 in mice is associated with retinal degeneration [25]. In GBM, CD133 is important for the maintenance of GSCs. CD133 knockdown with shRNA in GBM cells leads to impaired tumorsphere formation *in vitro* and tumorigenicity *in vivo* [26]. Phosphorylation of the Y828 tyrosine residue in the cytoplasmic C-terminus of CD133 leads to activation of the PI3K/Akt pathway within GSCs [27]. Detailed analysis of CD133-mediated signaling has been hampered by the fact that there are no known ligands.

Studying the cellular properties of CD133-expressing cells is crucial to furthering our understanding of GBM biology. In addition, due to its membrane localization, CD133 represents a good candidate for GSC-targeted therapeutics. Viral gene transfer is a popular and exciting technology for the genetic manipulation of cells *in vitro* and *in vivo*. Lentiviral vectors have attracted a lot of attention due to their ability to generate long-term stable gene delivery into a broad spectrum of cell types, independent of their cell cycle status [28], [29]. Cellular tropism of lentiviruses can be altered by their envelope proteins. Lentiviral vectors used for gene transfer have traditionally been engineered to express not their native envelope proteins, but rather proteins that facilitate transduction of target cells [30], [31]. The most widely used lentiviral envelope confers wide cellular tropism, by virtue of the G glycoprotein of the vesicular stomatitis virus (VSVG), which binds the LDL receptor of the host cell membrane [32]. Although wide tropism is useful for many laboratory applications, a refined selectivity is required for cell-type specific delivery. While cellular specificity can be conferred by tissue-specific promoters, such promoters are not always available or may be too large for proper vector packaging.

Anliker et al. (2010) recently described a new strategy for conferring selective tropism to lentiviral vectors by engineering single chain antibody sequences into the viral envelope [33]. One of their recombinant lentiviral vectors, CD133-LV, presents within the viral envelope a single chain antibody against the 293C3 epitope on the second extracellular loop of human CD133 (**Fig. 1A,B**) [33]. The antibody sequence was fused to a mutated hemagglutinin protein of the measles virus to allow for pH-independent viral entry into target cells. The fusion envelope protein confers restricted tropism to CD133-expressing cells via the interaction of the antibody sequence to its cognate antigen on the target cell surface. Furthermore, four point mutations in hemagglutinin prevent cell entry via the natural measles virus receptors. The resulting CD133-LV is able to selectively transduce CD133+ human hematopoietic progenitor cells, which are poorly transduced with pantropic VSVG-pseudotyped lentiviral vectors [33].

Here, we present CD133-LV as a highly selective gene delivery system to CD133-expressing GSCs. We show that CD133-LV selectively transduces CD133+ GSCs in primary human GBM cultures and intracranial GBM xenografts in NOD.SCID mice, while human embryonic stem cell (hESC)-derived neurons, primary human astrocytes and normal mouse brain tissue remain uninfected. We propose that CD133-LV represents a valuable tool for the selective transduction of CD133+ GSCs and that the technology of engineering antibodies into the envelope protein of lentiviral vectors can be applied to the study of other cell types with unique surface markers in brain and systemic tumors.

## Materials and Methods

### Primary tumor cultures and cell lines

We followed a protocol approved by NYU Langone Medical Center's Institutional Review Board (IRB) to procure fresh tumor tissue from patients undergoing surgery for resection of GBM (IRB# S12-01130). Within 1–2 hours after surgical resection, tumor specimens were minced in Hank's Balanced Salt Solution (HBSS, Life Technologies) and enzymatically dissociated into single cells (Accutase, Innovative Cell Technologies). Upon dissociation, cells were cultured in suspension on non-adherent plates in Neurobasal media (Life Technologies), supplemented with N2 (Life Technologies), B27 (without vitamin A; Life Technologies), 20 ng/mL of human recombinant EGF (Life Technologies) and 20 ng/ml bFGF (R&D Systems) [34]. Four primary GBM cultures were used for the experiments described here. Viral transduction efficiency and selectivity were assessed on GBML3, GBML8 and GBML20 cultures. GBML8 and GBML20 cultures were used for CD133 knockdown. GBML3 and GBML27 cultures were used for CD133 overexpression experiments.

Primary melanoma cells (NYU10-230) were cultured in Roswell Park Memorial Institute (RPMI) 1640 Medium (Life Technologies) supplemented with 10% fetal bovine serum (FBS, Life Technologies) and non-essential amino acids (Life Technologies) [35].

U87-MG cells for production of intracranial xenografts, Lenti-X 293 HEK cells (Clontech) for lentivirus production and Huh7 cells for viral titering were cultured in Dulbecco's Minimal Essential Media (DMEM, Life Technologies) supplemented with 10% FBS and non-essential amino acids.

Primary normal human astrocytes (Lonza) were cultured using Astrocyte Growth Media Bullet kit (Lonza), as in the manufacturer's protocol.

### Human embryonic stem cell culture and neuronal differentiation

H9 (WA09) hESCs were cultured as previously described [36]. Briefly, undifferentiated pluripotent hESC colonies were grown on a feeder layer of mouse embryonic fibroblasts (MEFs) in DMEM/F-12 medium supplemented with 20% knockout serum replacement (Life Technologies) and 5 ng/ml bFGF (**S1A,B Fig.**).

A two-step approach was utilized to obtain hESC-derived neurons (**S1C Fig.**). In the first step, hESC colonies were placed in suspension culture for 10 days as serum-free embryoid bodies (SFEBs) in media supplemented with 100 ng/ml noggin (R&D Systems), a peptide neutralizing BMP ligands, 200 nM LDN193189 (Stemgent), an inhibitor of BMP type I receptors, and 1 µM SB431542 (Tocris Biosciences), an inhibitor of TGFβ type I receptor. SFEBs were plated on polyornithine (PO)/laminin-coated dishes for an additional 4 days to produce rosette-type multipotent neural precursors (**S1D Fig.**). In the second step, rosettes were picked, replated on PO/laminin-coated dishes and cultured for 2 weeks in the presence of Neurobasal media supplemented with N2 (Life Technologies), 20 ng/ml brain-derived neurotrophic factor (BDNF; R&D Systems) and 0.2 mM ascorbic acid (AA; Sigma) [37]. This differentiation protocol resulted in formation of differentiated neurons identified by immunostaining for the dendritic marker MAP2A.

### Lentivirus production

Lentiviruses were generated in Lenti-X 293 HEK (Clontech) producer cells after transfection with a combination of: transfer plasmid, encoding the viral genome; packaging plasmids, encoding structural and enzymatic components of viral particles; and envelope plasmids, encoding viral envelope proteins. The architecture of the viral genomes employed in this study is shown in **S2A Fig.** The promoters and transgenes used are listed in **S2B Fig.** Envelope and packaging plasmids are shown in **S3 Fig** **.**

CD133-LV was packaged using the relevant transfer plasmid along with the following plasmids: pLP1, pLP2, pHnseL3-scFv141.7, pCG-Fnse-d30 [33]. VSVG-LV was assembled using a transfer plasmid along with: pLP1, pLP2, pLP-VSVG.

Lentiviral vectors for expression of shRNA or overexpression of *PROM1* cDNA were assembled similar to VSVG-LV.

Lentiviral vectors were produced in Lenti-X 293T HEK cells after transfection of plasmids with Lipofectamine-2000 (Life Technologies). Lentiviral supernatant was collected at day 2 and 3 after transfection, filtered (0.45 µm filter) and concentrated with ultracentrifugation (28,000 g for 3 hours at 4°C) using a 4% sucrose/PBS cushion. After centrifugation, the supernatant was discarded and viral pellets were resuspended in Opti-MEM medium, aliquoted and stored at -80°C.

For lentiviral vectors expressing fluorescent proteins, titers were determined by transduction of either 293T cells (in the case of VSVG-LV) or Huh7 cells (in the case of CD133-LV), and measurements by flow cytometry. For lentiviruses that did not express fluorescent transgenes, we determined their titers by qPCR-based assays (ABM).

### Viral transduction

Primary GBM tumorsphere cultures were dissociated with Accutase (Innovative Cell Technologies). 30,000 cells were incubated at 37°C for 4 hours with either CD133-LV or VSVG-LV at various multiplicity of infection (MOI) ratios in a 50 µl volume. Human melanoma cells, neurons and astrocytes were plated at a density of 30,000 cells/well in 24-well plates, and viral transductions were performed at 37°C for 4 hours in a 200 µl volume. Protamine sulfate (4 µg/mL) was added to facilitate viral transduction. Transduction efficiency was analyzed 3 days after transduction with either flow cytometry using the LSRII analyzer (BD Biosciences) or immunofluorescent microscopy. Enrichment of CD133+ cells in the transduced cell fraction was calculated using the following formula: *enrichment = observed/expected fraction*, where the expected fraction represents the prevalence of CD133+ cells within the parent culture.

### CD133 overexpression and shRNA-mediated CD133 knockdown

To constitutively overexpress CD133 in human GBM cells, we generated a lentiviral vector (CD133-OE) expressing *PROM1* under the control of the eukaryotic EF1α promoter (**S2B Fig.**) [26]. In order to knock down CD133 expression in human GBM cells, we modified vector pLKO.1 (Addgene plasmid 10878) to express an shRNA (5′-GGCTAGATTCTAACATATT-3′) targeting the 3′-UTR of *PROM1*. (**S2B Fig.**) [38]. We also constructed a control vector expressing a scramble shRNA sequence (5′-CCTAAGGTTAAGTCGCCCTCG-3′) with no expected human mRNA targets.

### Flow cytometry

For flow cytometric analysis, cells were dissociated with Accutase. CD133 staining was performed with fluorophore-conjugated AC133 antibody, which recognizes the CD133/1 epitope, or 293C3 antibody, which recognizes the CD133/2 epitope (Miltenyi). Both epitopes reside in the second extracellular loop of CD133. AC133 antibody was used for surface expression analysis and enrichment analysis upon viral transduction. 293C3 antibody was used to assess surface protein levels upon knockdown and overexpression of CD133. The LSRII analyzer (BD Biosciences) was used for flow cytometric measurements. For fluorescence-assisted cell sorting (FACS), a FACSAria cell sorter (BD Biosciences) was used with assistance from the NYU Langone Medical Center's Cytometry and Cell Sorting Core Facility staff.

### Animals and stereotactic injections into mouse brain

Mice were housed within NYU Langone Medical Center's Animal Facilities. All procedures were performed according to our IACUC-approved protocol. 6–8 week old NOD.SCID (Jackson Laboratory, NOD.CB17-Prkdcscid/J, 001303) male mice were anesthesized with intraperitoneal injection of ketamine/xylazine (10 mg/kg and 100 mg/kg, respectively). They were then mounted on a stereotactic frame (Harvard Apparatus). A midline skin incision was made. A high-speed drill was used to drill a small hole in the calvaria 2 mm off the midline and 2 mm anterior to coronal suture. Five µl of a suspension of human GBM cells (100,000 cells/µl, unless otherwise noted) were injected through a Hamilton syringe (1 µl/min, Harvard apparatus, needle pump) into the frontal lobe through the drilled hole. The injection needle was left in place for an additional 5 minutes after the injection was completed to prevent backflow. The skin incision was sutured and animals were monitored throughout the recovery period. Tumor growth was monitored with small animal MRI.

For viral transduction of intracranial human GBM xenografts, 2 µl of high-titer lentivirus (∼10^8^ TU/ml) were injected into the tumor via stereotactic coordinates obtained by MRI. Injection was performed with a heat-pulled glass capillary using a pressurized injector (PicoPump, World Precision Instruments). The glass needle was left in place for an additional 5 minutes after injection to prevent backflow. Animals were sacrificed 1 week after virus injection to analyze transduction by flow cytometry and immunofluorescence confocal microscopy.

### Small animal MRI

Tumor formation was analyzed 1.5 months (unless otherwise noted) after injection of tumor cells into the brains of NOD.SCID mice. An MRI device bearing a 7-Tesla horizontal bore Bruker magnet (ID = 300 mm with zero boil off technology) in the Small Animal Imaging Core Facility at NYU School of Medicine was used for imaging. Prior to imaging animals were anesthetized with isoflurane gas. Stacked images were processed using ImageJ software. Tumor volumes were calculated with Amira Software.

### Immunofluorescence staining and microscopic analysis

When the experimental end-point was reached, animals were anesthetized with Ketamine/Xylaxine (10 mg/kg and 100 mg/kg, respectively) and systemically perfused with first Phosphate-buffered Saline (PBS) and then 4% paraformaldehyde. Isolated brain tissue was mounted in OCT (Tissue-Tek) and 30 µm-thick frozen sections were obtained using a cryostat (Leica). Sections were blocked with 10% (w/v) BSA, 0.1% Triton X-100 in PBS (BSA; Sigma) for 2 hours at room temperature. The following primary antibodies were used for immunostaining: anti-CD133 (AC133 clone, Miltenyi), anti-DsRed (Clontech; used to detect mCherry), anti-RFP (Invitrogen; used to detect TagBFP) and anti-NeuN (Abcam). Staining was performed in blocking solution for 18 hours at 4°C.

Human ESC cultures, rosette-type neural precursor cells, neurons and astrocytes were fixed with 4% paraformaldehyde for 15 minutes followed by blocking in blocking solution (10% BSA, 0.1% Triton X-100 in PBS) for 1 hour. All antibody incubations were done for 18 hours in blocking solution at 4°C. The following primary antibodies were used for immunostaining: anti-Nanog (R&D Systems), anti-Oct3/4 (Santa Cruz), anti-Sox2 (R&D Systems), anti-ZO1 (Invitrogen), anti-PLZF (Santa Cruz), anti-MAP2A (Millipore) and anti-GFAP (Dako).

Alexa488, Alexa555 and Alexa647 - conjugated secondary antibodies were used for fluorescent labeling (Life Technologies). Nuclear chromatin was counterstained with DAPI (Sigma). Epifluorescence microscopy was performed on a Eclipse E800 fluorescent microscope (Nikon). For confocal imaging, 30 µm z-stacks were obtained with an LSM700 confocal microscope (Zeiss). Image analyses were performed on ImageJ and Adobe Photoshop.

### Western Blotting

Primary GBM cells were lysed in Lysis Buffer (150 mM NaCl, 50 mM Tris pH 7.4, 1 mM EDTA, 0.1% Triton-X100, 10% glycerol) supplemented with complete protease inhibitor cocktail (Roche). Lysates were centrifuged to remove debris and the supernatant was quantified using the Bradford assay. The supernatant was separated on an SDS-PAGE gel and transferred to a nitrocellulose membrane (Biorad). The membrane was probed with the following primary antibodies: anti-CD133 (Miltenyi) and anti-β-Actin (Santa Cruz Biotechnology). Signal was detected with appropriate secondary antibodies suited for either infrared fluorescence (Licor) or chemiluminescence (Thermo Scientific).

### Statistical Analysis

Statistical comparisons included Student's unpaired two-tailed t-test; and one-way and two-way analysis of variance (ANOVA), followed by *post hoc* analysis with Tukey's test. Statistical significance cutoff was set at p<0.05. SPSS software (IBM) was used for statistical analyses. Population statistics were represented as mean ± standard error (SE) of the mean.

### Ethics Statement

Tumor tissue was collected from patients undergoing surgery for GBM resection at NYU Langone Medical Center after written informed consent and in compliance with a protocol approved by the Institutional Review Board (IRB# S12-01130). NYU Langone Medical Center's IRB specifically approved this study.

Animal experiments were carried out in accordance with a protocol approved by NYU Langone Medical Center's Institutional Animal Care and Use Committee (IACUC# 120310-03). All surgery was performed under Ketamine/Xylazine anesthesia as described above and all efforts were made to minimize suffering.

## Results

### Primary cultures from human GBM biospecimens and characterization of CD133+ GSCs

To establish primary human GBM tumorsphere cultures, we have developed a protocol for the transfer of human GBM biospecimens from the operating room to the laboratory. We generated primary GBM cultures, as previously described [34]. Briefly, upon dissociation, tumor cells were cultured in suspension with EGF and FGF (20 ng/ml each) and allowed to form tumorspheres. Once primary sphere cultures had been established, we measured the relative number of CD133+ cells by flow cytometry (**Fig. 1C** **i,ii, S4A Fig.**).

The primary lines used in this study have been in culture for varying times ranging from twelve months to more than two years (**S4A Fig.**). Although CD133+ cells were present in all cultures, their relative abundance varied among the four primary cultures (29.5±19.3%, n = 4) (**S4A Fig.**). After establishment of tumorsphere cultures, the abundance of CD133+ cells remains constant over time (**S4B Fig.**), suggesting that these cells self-renew in serum-free tumorsphere culture conditions. To further assess the stem cell characteristics of CD133-expressing GBM cells, we FACS-isolated CD133+ and CD133-cells and compared them with qRT-PCR. We observed enrichment of the GSC-associated transcripts *PROM1*, *Nestin* and *Sox2* in the CD133+ fraction of primary cultures (GBML3, **S4C Fig.**). Furthermore, FACS-isolated CD133+ GBM cells gave rise to larger intracranial tumors compared to their CD133- counterparts, when injected in equal numbers (15,000 cells/animal) into NOD.SCID mice (**S4D Fig.**). Collectively, these data suggest that CD133+ cells in our primary GBM cultures represent GSCs.

### CD133-LV transduces primary human GBM cultures in dose-dependent manner

To determine the efficiency by which CD133-LV transduces human GBM cells, we generated a fluorescent version of CD133-LV. We introduced the blue fluorescent protein TagBFP under the control of the constitutive SFFV promoter to facilitate the identification of transduced cells. To test the transduction efficiency in GBM cells, we dissociated tumorspheres to single cell suspensions and incubated them with CD133-LV (MOI = 5) (**Fig. 1C** **iii**). As a control, we also transduced the human GBM cell suspension with a non-selective pantropic VSVG-LV at the same MOI (**Fig. 1C** **iv**), before we measured the relative transduction efficiencies by flow cytometry (**Fig. 1C** **iii,iv**). As expected, we observed that the CD133-LV transduction efficiency increased with the number of CD133-expressing GBM cells contained in the parent culture (**Fig. 1D**), while VSVG-LV resulted in uniformly high transduction efficiencies independent of the CD133 content of the culture (**Fig. 1C** **iv**). Cumulatively, at this MOI (MOI = 5), CD133-LV transduced only a small number of cells (6.1±1.7%, n = 3 primary lines), compared to pantropic VSVG-LV (73.8±5.3%, n = 3 primary lines). (**Fig. 1C** **iii,iv**). Live fluorescence microscopy of tumorspheres transduced with CD133-LV showed scattered cells expressing TagBFP 3 days after incubation with CD133-LV, confirming transduction (**Fig. 1E**).

To directly compare the transduction efficiencies of CD133-LV and VSVG-LV, we performed titration experiments using MOIs ranging from 0.16 to 10 in one of our primary GBM cultures, GBML3, which contains 1.7±0.1% CD133+ cells. Transduction efficiencies of CD133-LV and VSVG-LV were both dose-dependent (ANOVA F_9,19_ = 5.116, P<0.001; and F_9,20_ = 22.454, p<10^-8^, respectively) (**Fig. 1F**). Pantropic lentivirus VSVG-LV gave significantly higher transduction rates throughout the range of doses tested (ANOVA F_1,29_ = 24.564, P<0.0001) (**Fig. 1F**).

Collectively, these findings indicated that CD133-LV successfully transduced a fraction of human GBM cells in a number of primary cultures *in vitro*.

### CD133-LV selectively transduces CD133+ cells *in vitro*

**Figure 2:**
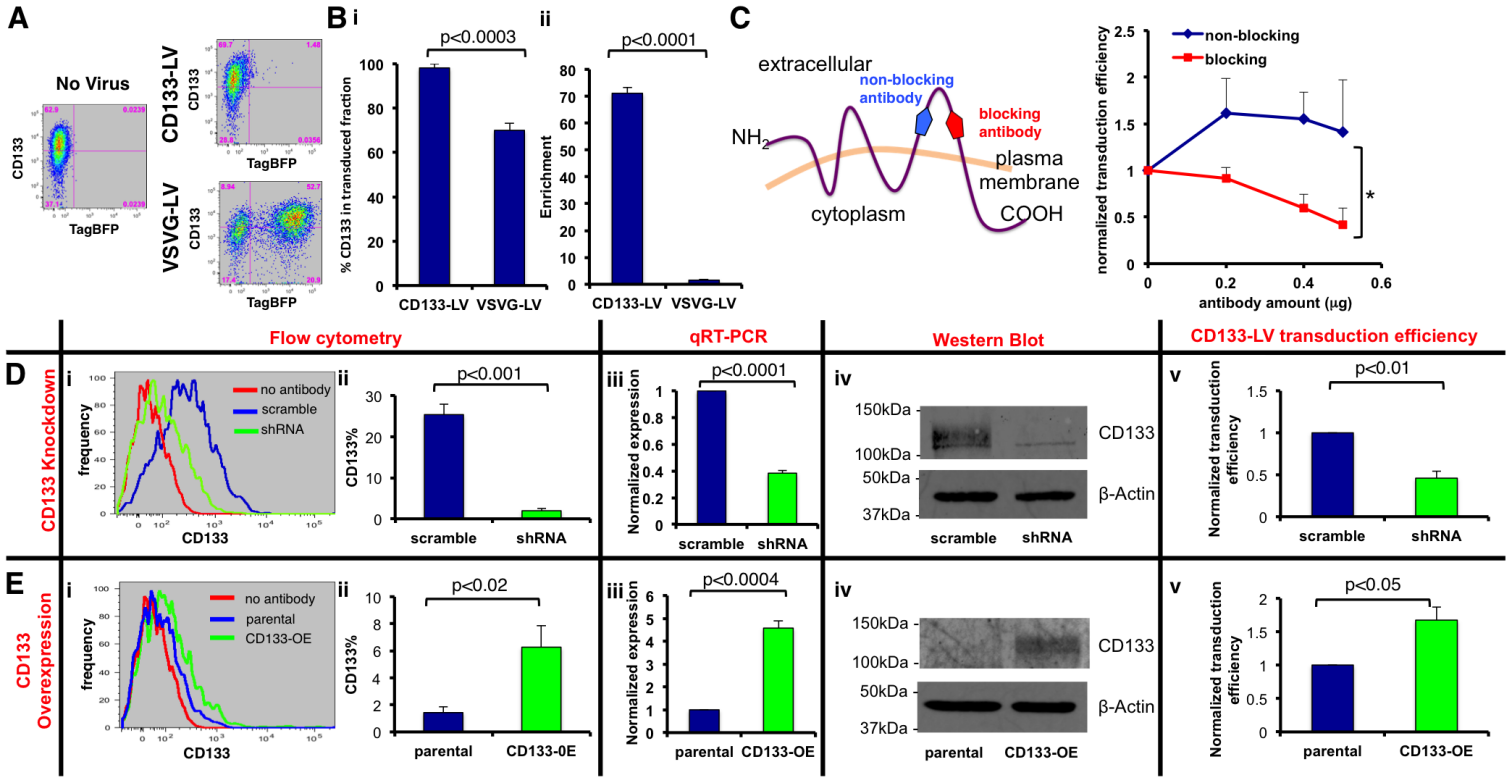
Selectivity of transduction by CD133-LV. **A**. Flow cytometry analysis with GBML20 (CD133 content is 68.4±9.8%) shows that CD133-LV - transduced cells (TagBFP+) are also positive for CD133 (right top). In contrast, CD133+ cells are not enriched in the cohort transduced with VSVG-LV (right bottom). Untransduced cells show no TagBFP expression as expected (left panel) **B**. **i**. Percent CD133 positivity of cells transduced with either CD133-LV or VSVG-LV. **ii**. Population statistics for enrichment of CD133+ cells within populations transduced by either CD133-LV or VSVG-LV (MOI = 1). **C**. Schematic representation of epitopes on the second extracellular loop of CD133 recognized by antibodies predicted to block (recognizing 293C3 epitope) or not block (recognizing AC133 epitope) the interaction of CD133-LV's envelope with CD133 on the cell surface. Primary GBM cells were treated with varying amounts of blocking or non-blocking antibody prior to transduction with CD133-LV (MOI = 0.5). Transduction efficiency of CD133-LV was significantly reduced with blocking antibody. In contrast, non-blocking antibody did not show any significant effect (*, p<10^−7^). **D,E**. Primary GBM lines were modified with lentiviral constructs to either knockdown (**D**) or overexpress CD133 (**E**). GBML20 (CD133 content 68.9±9.8%) was used for shRNA-mediated knockdown of CD133. GBML27 (CD133 content 1.4±0.4%) was used for CD133 overexpression after transduction with lentiviral vector CD133-OE. **i-ii**. Flow cytometric analysis showing the CD133 content of primary lines expressing shRNA against CD133 or overexpressing CD133. **iii**. qRT-PCR analysis confirmed knockdown and overexpression of *PROM1* mRNA. **iv**. Western Blotting confirmed knockdown and overexpression of CD133 in these lines. β-actin was used as loading control. **v**. CD133 knockdown in GBML20 led to reduced transduction with CD133-LV (MOI = 5). Conversely, CD133 overexpression in GBML27 increased the rate of transduction by CD133-LV (MOI = 5).

We performed further experiments to test the hypothesis that CD133-LV selectively transduces CD133+ GBM cells *in vitro*. We transduced primary human GBM cultures (GBML20 whose CD133+ content is 68.4±9.8%) with TagBFP-expressing CD133-LV or VSVG-LV to measure the expression of TagBFP amongst CD133+ GBM cells by flow cytometry 3 days after infection (**Fig. 2A**). Our analyses revealed that 98.2±1.8% of CD133-LV - transduced GBML20 cells were CD133+. In contrast, GBML20 cells transduced with VSVG-LV showed high transduction rates with no predilection for the CD133+ cohort (70.0±3.2% CD133+ cells among transduced cohort, t-test, p<0.0003 n = 3) (**Fig. 2A** **, Bi**). Indeed, the enrichment (*enrichment = observed/expected fraction*) of CD133+ cells among TagBFP+ transduced cells was 71.2±2.0 -fold, as opposed to 1.6±0.2 -fold in VSVG-LV - infected GBML20 cells (t-test, p<0.0001, n = 3) (**Fig. 2B** **ii**), suggesting that CD133-LV has restricted tropism toward CD133+ cells.

To confirm the findings with GBML20, we repeated the same experiments with GBML8 (CD133 content is 46.5±5.7%) (**S5A Fig.**). Similar to GBML20, we observed that 94.9±0.4% of CD133-LV - transduced cells in GBML8 were CD133+ (n = 3), whereas CD133+ cells represented only 44.1±2.7% of all VSVG-LV - transduced cells (n = 3; t-test, p<0.0001) (**S5B Fig.**). We found significant enrichment of CD133+ cells within the TagBFP+ transduced population with CD133-LV (20.0±7.4 –fold as opposed to 1.1±0.0 with VSVG-LV; t-test, p<0.02, n = 3) (**S5C Fig.**).

The selectivity of CD133-LV depends on the interaction of the CD133 antibody on the viral envelope and the CD133 epitope on the cell surface [33]. To show that transduction is mediated by this interaction, we treated our GBM cells with increasing amounts of an antibody recognizing the same epitope as the CD133-LV viral envelope protein (293C3, blocking antibody), or with an antibody that recognizes a different epitope on CD133 (AC133, non-blocking antibody) and measured transduction efficiency with CD133-LV by flow cytometry (**Fig. 2C**). These experiments revealed a more than 50% reduction in transduction efficiency with CD133-LV upon treatment with blocking antibody (ANOVA F_1,15_ = 100.276, p<10^−7^), while treatment with non-blocking antibody had no significant effect on transduction efficiency (**Fig. 2C**).

If CD133-LV is selectively tropic toward CD133+ GBM cells, it follows that increasing or decreasing the level of CD133 expression in primary cultures should affect transduction efficiency accordingly. To test this hypothesis, we modified the CD133 content of primary GBM lines with lentiviral constructs either overexpressing *PROM1* cDNA or expressing shRNA constructs targeting *PROM1* mRNA transcripts.

GBML20, a primary culture with high CD133+ cell content (68.4±9.8%, n = 3 measurements) was transduced with a lentivirus expressing an shRNA against CD133 (shRNA-LV). In a control experiment, we transduced GBML20 cells with a lentivirus expressing a scramble shRNA sequence with no predicted mRNA targets (**Fig. 2D**). Flow cytometry of GBML20 cells modified to express shRNA or scramble shRNA indicated that the percentage of CD133+ cells decreased dramatically to 2.0±0.6% (n = 3) when shRNA against CD133 was expressed, but remained high (25.4±2.6%, n = 3), when the scramble shRNA sequence was used (t-test, p<0.001) (**Fig. 2D** **i,ii**). CD133 knockdown was further confirmed by qRT-PCR for *PROM1* transcript (**Fig. 2D** **iii**) and Western blot analyses of total protein lysates from transduced cultures (**Fig. 2D** **iv**). CD133 knockdown significantly reduced CD133-LV's transduction efficiency to 46.0±8.1% of the scramble condition as assayed by flow cytometry (n = 4 for each condition, MOI = 5; t-test, p<0.01) (**Fig. 2D** **v**), consistent with the idea that CD133-LV has selective tropism. Similar results were obtained with GBML8 cultures (CD133+ content 46.5±5.7%, n = 3 measurements) that were transduced with lentiviral constructs expressing shRNA against *PROM1*, further confirming selective tropism of CD13-LV (**S5D Fig.**).

To test whether ectopic expression of CD133 in a primary GBM line that contains only a small subset of CD133+ cells can increase the transduction efficiency of CD133-LV, we infected primary GBML27 cells, in which CD133+ cells represent only 1.4±0.4% (n = 5 measurements) of all cells, with a lentivirus that expresses *PROM1* cDNA under the constitutive EF1α promoter (CD133-OE construct) (**Fig. 2E**). Following transduction, we measured a relative increase in the abundance of CD133+ cells from 1.4±0.4% to 6.3±1.6% (n = 5; t-test, p<0.02) by flow cytometry (**Fig. 2E** **i,ii**). Likewise, qRT-PCR and Western blot analyses of total protein lysates from parental untransduced and CD133-OE - transduced cultures confirmed increased expression of *PROM1* transcript and protein (**Fig. 2E** **iii,iv**). Following CD133 overexpression, we measured a 67.5±0.2% increase in CD133-LV's transduction efficiency (n = 4 for each condition, MOI = 5; t-test, p<0.05) by flow cytometry. We have reproduced the increase in transduction efficiency of CD133-LV upon CD133 overexpression with GBML3 (baseline CD133+ content 1.7±0.1%, n = 5 measurements), further confirming viral selectivity (**S5E Fig.**).

Together, these experiments suggest selective tropism of CD133-LV towards CD133+ human GBM cells. Furthermore, the reproducibility of these results with different primary GBM cultures suggests global utility of CD133-LV in the genetic manipulation of CD133+ cells in GBM.

### Human GBM cells transduced with CD133-LV retain stem-like properties

**Figure 3:**
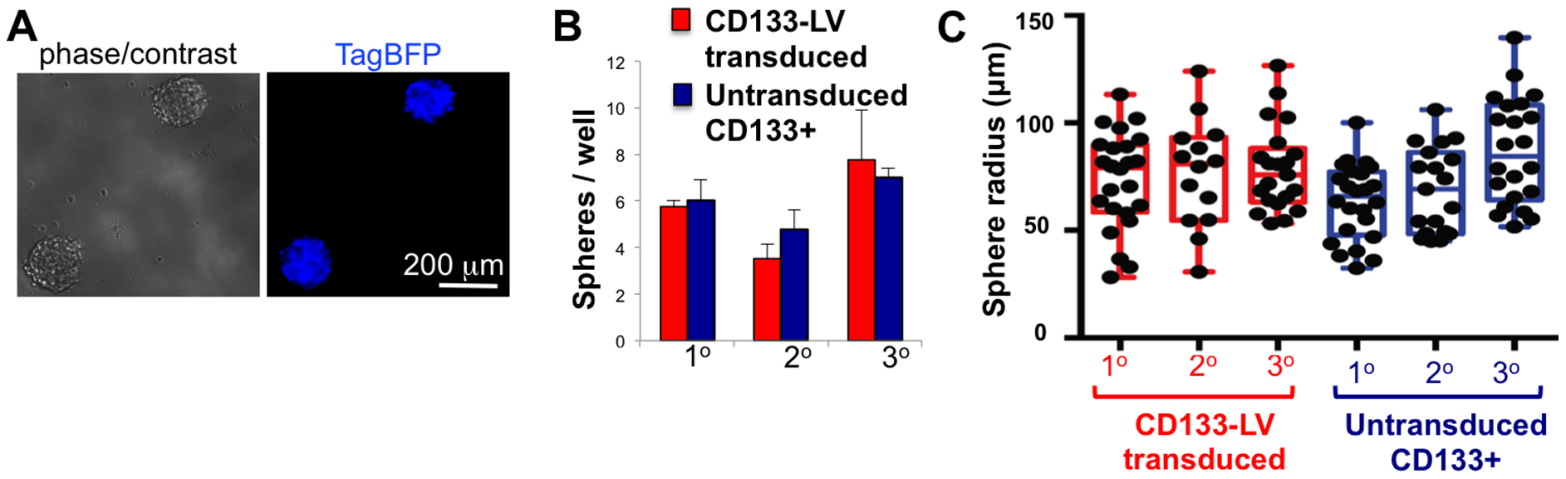
Stem-like properties of GBM cells transduced with CD133-LV. **A**. Cells transduced with CD133-LV expressing TagBFP are clonogenic and produce spheres comprised of TagBFP+ cells. **B,C**. *In vitro* tumorsphere formation assays for 3 serial passages did not show any difference in the clonogenic potential of FACS-isolated CD133-LV transduced cells and untransduced CD133+ cells in terms of the number (**B**) and size (**C**) of spheres formed.

Human GSCs have the ability to initiate the formation of tumorspheres *in vitro*. In order to assess whether human GBM cells transduced with CD133-LV have stem-like properties, we transduced our primary GBM cultures with CD133-LV, isolated them by flow cytometry based on their TagBFP expression, and plated them at low density (10 cells/µl) to measure their sphere-forming potential over serial passages. In parallel, we also determined the sphere-forming potential of untransduced CD133+ GBM cells, which we isolated by FACS using fluorescently coupled anti-CD133 antibodies. CD133-LV - transduced cells were clonogenic and gave rise to TagBFP+ tumorspheres (**Fig. 3A**). Furthermore, tumorspheres generated either from CD133-LV - transduced or untransduced CD133+ cells did not differ in terms of sphere numbers (**Fig. 3B**) or their size distribution (**Fig. 3C**) over 3 serial passages. This finding suggests that CD133-LV transduces human GBM cells with stem-like properties and that primary GBM cells retain their stem-like characteristics when transduced with lentivirus.

### CD133-LV transduces CD133+ human GBM cells *in vivo*

**Figure 4:**
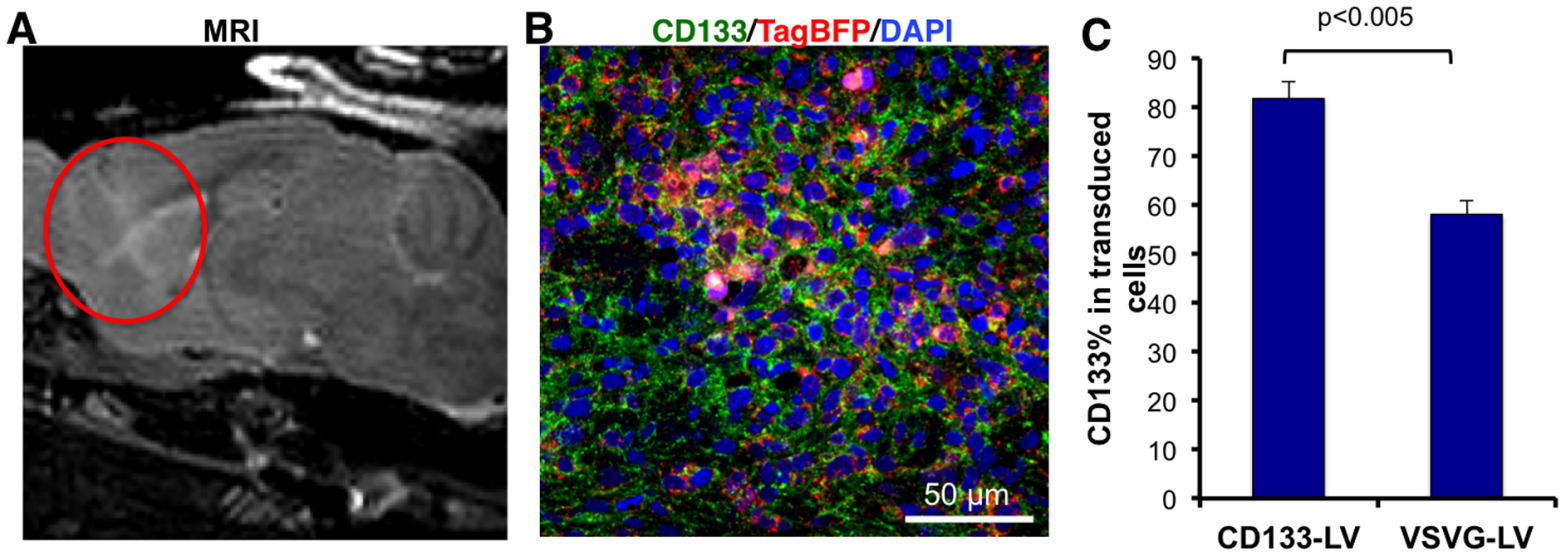
CD133-LV transduces CD133+ cells in primary GBM xenografts in the mouse brain. **A**. Intracranial xenograft tumors were generated using injection of GBML20 cells (5×10^5^ cells/animal) and tumor formation (red circle) was confirmed with small animal MRI 1.5 months after injection. High titer stocks of CD133-LV or VSVG-LV expressing TagBFP were injected into the tumor and animals were sacrificed 7 days later for immunofluorescence analysis. **B**. CD133-LV - transduced cells expressing TagBFP (red) show cell surface immunoreactivity for CD133 (green). **C**. CD133+ cells were significantly more enriched among TagBFP+ transduced cells in the case of CD133-LV compared to VSVG-LV.

In order to assess the transduction efficiency of CD133-LV *in vivo*, we established intracranial human GBM xenografts (CD133 content: 71.7±8.7%, n = 3 tumors generated by injection of GBML20) in NOD.SCID mice. Tumor growth was confirmed with MRI. Using stereotactic coordinates inferred from MRI images, we stereotactically injected TagBFP-expressing high-titer CD133-LV or VSVG-LV (10^8^ TU/ml) into intracranial tumors (**Fig. 4A**). Seven days after viral infection we sacrificed the mice and analyzed the xenograft by confocal microscopy and flow cytometry. Our microscopic analyses identified TagBFP-expressing CD133+ human GBM cells (**Fig. 4B**). Likewise, our flow cytometric analyses of transduced tumors identified 81.8±3.5% (n = 3) CD133+ cells within the TagBFP+ CD133-LV - transduced cohort, while only 58.1±2.7% (n = 3) CD133+ cells were detected within the TagBFP+ cohort when xenografts were infected with VSVG-LV (t-test, p<0.005) (**Fig. 4C**).

We assessed selectivity of CD133-LV *in vivo* with two additional orthotopic xenograft models. Xenografts generated with GBML8 (CD133 content: 46.5±5.7%) were injected with high-titer CD133-LV (10^8^ TU/ml) expressing TagBFP. Immunofluorescence analysis of the tumors 7 days after viral injection revealed CD133+ cells with TagBFP expression suggesting selectivity *in vivo* (**S6A Fig.**). Additionally, established GBM cell line U87-MG (CD133 content: 0.1±0.0%, n = 5) was used to generate intracranial tumors in NOD.SCID animals. Upon identification of tumors with MRI, high-titer CD133-LV or VSVG-LV expressing mCherry (10^8^ TU/ml) was injected into the tumors. CD133-LV led to very low transduction *in vivo*, consistent with the low abundance of CD133 in U87-MG xenografts, as opposed to VSVG-LV, which led to high transduction rates, as expected (**S6B Fig.**).

These *in vivo* findings are consistent our *in vitro* data experiments and suggest selective tropism of CD133-LV toward CD133+ human GBM cells. The fact that CD133+ cells did not represent an even higher percentage of the transduced population upon CD133-LV transduction *in vivo* may be explained by asymmetric division of CD133+ GSCs and generation of CD133- tumor cells that retained TagBFP expression.

### CD133-LV does not transduce normal mouse brain, human neurons or human astrocytes

**Figure 5:**
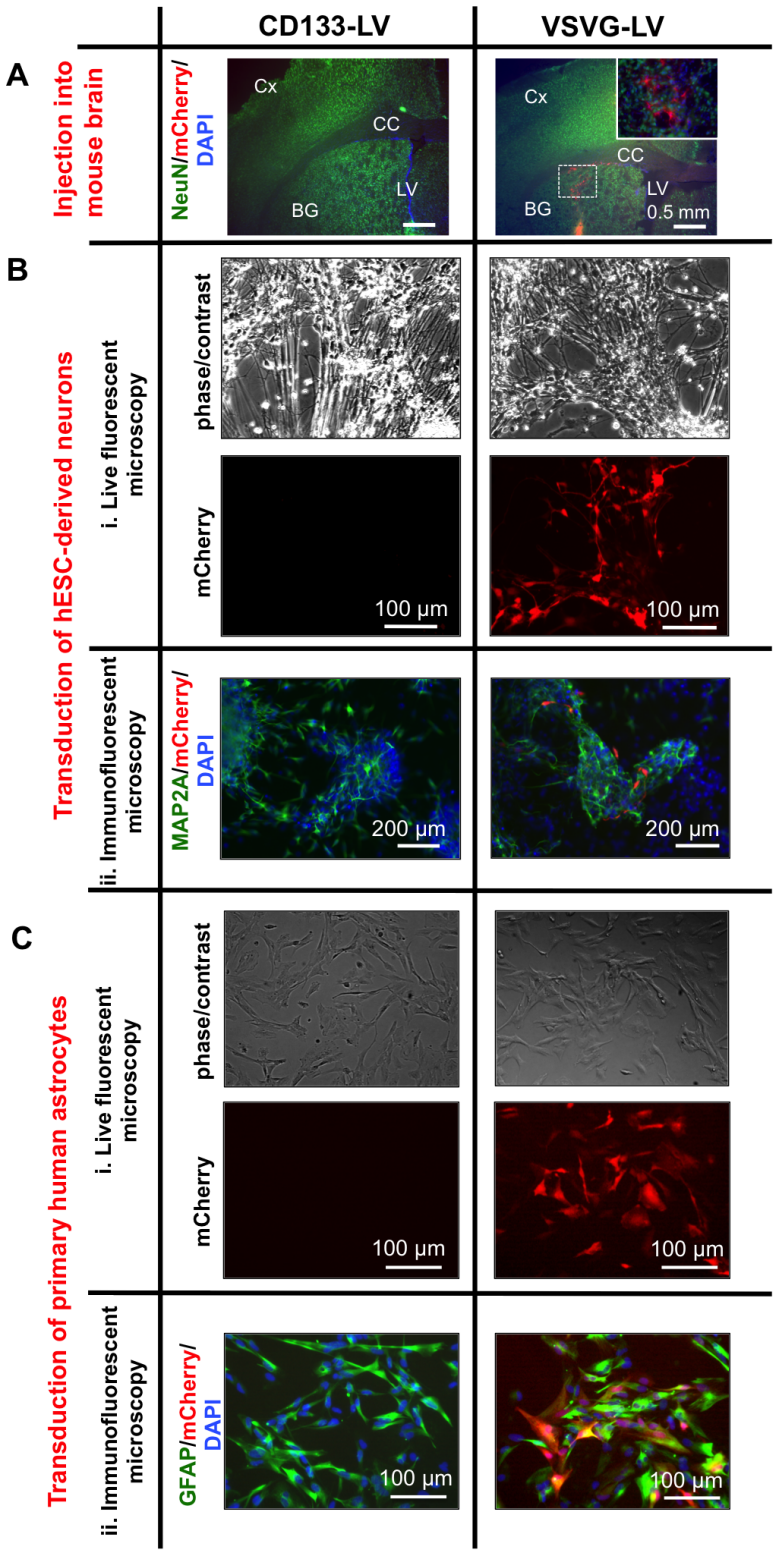
CD133-LV does not transduce normal mouse brain cells, hESC-derived neurons and primary human astrocytes *in vitro*. **A**. Injection of CD133-LV expressing mCherry into the mouse basal ganglia did not lead to transduction of normal brain tissue, as opposed to VSVG-LV (BG: basal ganglia, Cx: cortex, CC: corpus callosum, LV: lateral ventricle). **Bi,ii**. hESC-derived neurons were transduced with either CD133-LV or VSVG-LV expressing mCherry. VSVG-LV led to transduction of MAP2A+ neurons, as opposed to CD133-LV. **Ci,ii**. Primary human astrocytes were transduced with either CD133-LV or VSVG-LV expressing mCherry (MOI = 10). VSVG-LV led to transduction of GFAP+ astrocytes, as opposed to CD133-LV. (NeuN: neural nuclei, MAP2A: microtubule associated protein 2A, GFAP: glial fibrillary acidic protein). Nuclei were counterstained with DAPI.

Our experiments are geared towards selectively modifying CD133+ GSCs *in vitro* and *in vivo* in human GBM xenografts in the mouse brain. Adult mouse brain is known to express CD133 in the subventricular zone and ependymal cells, but not in neurons or glial cells [16]. In order to further analyze CD133-LV's selectivity and test the prediction that it does not transduce normal mouse neurons or glia, we performed the following experiment. We injected either CD133-LV or VSVG-LV expressing the red fluorescent protein mCherry into the basal ganglia of normal mouse brains. We observed no transduction with CD133-LV, as opposed to VSVG-LV, which led to widespread transduction along the injection site (n = 3) (**Fig. 5A**), as expected.

The CD133-LV envelope protein contains an antibody against human CD133. Using *in silico* analysis with NCBI Blast and algorithms to predict membrane topology (TMHMM 2.0), we found that mouse and human CD133 proteins are 53% identical and 76% similar in their amino acid sequences in the 2^nd^ extracellular loop, which contains the epitope that CD133-LV recognizes. Therefore, the lack of transduction of normal mouse brain neurons and glia by CD133-LV may be due to species incompatibility.

To further assess the selectivity of CD133-LV and to eliminate the issue of species compatibility, we tested whether CD133-LV transduces human neurons and astrocytes, which are not expected to express CD133. Human neurons were derived from hESCs in the presence of BDNF and AA (**S1 Fig** **.**) were transduced with equal titers of either CD133-LV or VSVG-LV expressing mCherry. We did not observe any transduction with CD133-LV as expected. In contrast, VSVG-LV transduced human neurons expressing the dendritic marker MAP2A (**Fig. 5B** **i**,**ii**). Primary human astrocytes were also transduced with equal titers of CD133-LV or VSVG-LV expressing mCherry. Similar to the results obtained with human neurons, no transduction was observed with CD133-LV but high transduction rates were obtained with VSVG-LV in GFAP-expressing human astrocytes (**Fig. 5C** **i**,**ii**). These findings confirmed that CD133-LV is selectively tropic to CD133-expressing human GBM cells and does not transduce human neurons or astrocytes non-specifically.

### CD133-LV selectively transduces CD133+ human melanoma cells *in vitro*

The above findings indicate that CD133-LV selectively transduces CD133+ human GBM cells and that transduction efficiency within the CD133+ population is low. To examine whether CD133-LV has similar transduction properties when used with other tumor types, we measured the transduction efficiency on a primary human melanoma line (**S7A,B Fig.**). We first measured the abundance of CD133+ cells in this culture by flow cytometry. We found that the relative abundance of CD133+ cells was 73.6±7.9% (n = 3). We then infected the cells with either CD133-LV or VSVG-LV expressing TagBFP at various MOIs. We found that CD133-LV transduced human melanoma cells at significantly lower rates than VSVG-LV (ANOVA F_2,6_ = 231.3, P<0.0001). Furthermore, CD133+ cells were 5.1 ± 0.7-fold enriched among CD133-LV – transduced cells (n = 3, MOI = 5), compared to only 0.9 ± 0.1-fold among VSVG-LV – transduced cells (n = 3, MOI = 5) (t-test, p<0.005). These results indicate that CD133-LV behaves similarly with human GBM and melanoma cells and selectively transduces CD133+ human tumor cells.

## Discussion

Collectively, our data suggest that CD133-LV selectively transduces CD133+ GSCs *in vitro* and *in vivo*, but spares hESC-derived neurons, human astrocytes and normal mouse brain tissue. However, a caveat to CD133-LV is its relatively low rate of transduction of CD133+ cells, a limitation replicated with GBM and melanoma cultures.

Although CD133 has been extensively used as a marker to isolate GSCs, the identity of CD133+ GSCs and how they contribute to GBM biology and therapy resistance remains unclear. Upon treatment with chemotherapeutic drugs, the abundance of CD133+ GBM cells increases, along with other GSC markers [39]. Furthermore, CD133+ GSCs are relatively resistant to radiation therapy *in vivo* [11]. Research on CD133+ GSCs has so far relied on FACS isolation on the basis of cell surface expression of CD133. However, the lack of appropriate lineage tracing technology has precluded the characterization of cellular progeny deriving from CD133+ GSCs in the context of tumor progression, therapy resistance and tumor recurrence *in vivo*.

Here, we present a CD133-tropic lentiviral vector, CD133-LV, which was previously shown to selectively target human hematopoietic progenitor cells, as a selective gene delivery method for CD133+ GSCs *in vitro* and in an intracranial mouse xenograft model *in vivo*. Previous data on hematopoietic cells and our data with primary human GBM and melanoma cultures show that CD133-LV is highly selective for human CD133+ cells [40]. We were able to selectively transduce CD133+ GBM cells *in vitro* and *in vivo*. We also observed that transduced cells maintain their sphere-forming potential without any appreciable cytotoxicity, suggesting that viral transduction does not interfere with any signaling pathway or transcriptional program required for stem cell function.

We propose that CD133-LV represents a promising tool for the selective genetic manipulation of human CD133+ GBM cells. We envision CD133-LV as a highly specific method for lineage trace analyses and cell type-specific ablation studies. Furthermore, CD133-LV could also be used to deliver transgenes or shRNAs specifically to CD133+ GSCs.

Using CD133-LV for therapeutic applications is an enticing possibility. Examples of such applications include selective delivery of cytotoxic or pro-differentiation genes to CD133+ GSCs, with the goal of eradicating them or suppressing their stem-like qualities, respectively. A significant current limitation in pursuing CD133-LV therapeutically is its relatively low transduction efficiency compared to pantropic lentiviral vectors. It is unclear which step in transduction is culpable for the lower efficiency with CD133-LV. Possible limiting steps include the binding of the virus to its cognate CD133 antigen on the cell surface, internalization, or the intracellular processing of the viral particle. Furthermore, GSCs may release CD133-containing membrane vesicles, which might lead to reduced transduction rates with CD133-LV, as occurs in the hematopoietic system [41]. Future research will aim to further define these steps and explore strategies to increase efficiency of transduction with CD133-LV. Bypassing the current limitations may one day allow the use of CD133-LV or similar viral vectors with selective tropism to be used therapeutically in GBM, or other brain and systemic tumors [42].

## Supporting Information

S1 Fig **Neuronal differentiation of hESCs.** **A**. Phase contrast microscopic images of hESC colonies grown on a layer of MEF feeders. **B**. Immunofluorescent microscopy shows that undifferentiated hESCs express the pluripotency-associated transcription factors Nanog, Oct3/4 and Sox2. **C**. The protocol utilized for generation of human neurons involves conversion of hESCs to rosette-type neural precursor cells, followed by neuronal differentiation in the presence of BDNF and AA. **D**. Rosettes show cell membrane immunoreactivity for the tight junction protein ZO1 (green) and transcription factor PLZF (red).
(PDF)

S2 Fig **Lentiviral genomes.** **A**. Architecture of the viral genomes used in this study (LTR: long terminal repeat, ψ: psi sequence, SD: splice donor, RRE: Rev response element, cPPT: central polypurine tract, WPRE: woodchuck hepatitis virus posttranscriptional response element, SIN3′-LTR: self-inactivating 3′-long terminal repeat). **B**. List of promoters and transgenes used in lentiviral vectors (SFFV: spleen focus-forming virus, CMV: cytomegalovirus, EF1α: eukaryotic elongation translation factor 1 alpha).
(PDF)

S3 Fig **Envelope and packaging plasmids for the production of lentiviral vectors.** **A**. Envelope plasmids used for generation of CD133-LV. **B**. Envelope plasmid for VSVG-LV. **C**. Packaging plasmids (P_CMV_: CMV promoter, RRE: Rev response element, P_RSV_: Rous sarcoma virus promoter, pA: polyadenylation site).
(PDF)

S4 Fig **Primary GBM cultures from human biospecimens and characterization of CD133-expressing GSCs.** **A**. Table summary of 4 different primary GBM cultures used in this study. **B**. Stability of CD133% content of primary GBM cultures over a period of 11 months. **C**. CD133+ cells from GBML3 were FACS-isolated and subjected to qRT-PCR analysis for GSC-associated transcripts (*PROM1*, *Nestin* and *Sox2*). **D**. FACS-isolated CD133+ and CD133- cells from GBML8 were injected (15,000 cells/animal) into NOD.SCID mice brains (n = 3 and n = 2 respectively). MRI analysis 4 months after injection revealed that CD133+ cells gave rise to larger tumors (circles) compared to CD133- cells (arrows), suggesting enhanced tumorigenic potential.
(PDF)

S5 Fig ***In vitro*** **validation of CD133-LV selectivity in multiple primary GBM cultures.** **A**. Flow cytometry analysis with GBML8 (CD133 content 45.6±5.7%) shows that CD133-LV - transduced cells (TagBFP+) are also positive for CD133 (right top). In contrast, CD133+ cells are not enriched in the cohort transduced with VSVG-LV (right bottom). Untransduced cells show no TagBFP expression as expected (left panel) **B**. **i**. Percent CD133 positivity within the transduced fraction of GBML8 with CD133-LV and VSVG-LV (MOI = 1). **C**. Population statistics for enrichment of CD133+ cells within the populations transduced by either CD133-LV or VSVG-LV (MOI = 1). **D,E**. Primary GBM lines were modified with lentiviral constructs to either knock down (**D**) or overexpress (**E**) CD133. GBML8 (CD133 content 45.6±5.7%) was used for shRNA-mediated knockdown of CD133. GBML3 (CD133 content 1.7±0.1%) was used for CD133 overexpression after transduction with lentiviral vector CD133-OE. **i**. Flow cytometric analysis showing the CD133+ content of primary lines expressing shRNA against CD133 or overexpressing CD133. **ii**. qRT-PCR analysis confirmed knockdown and overexpression of *PROM1* mRNA. **iii**. Western Blotting validated knockdown and overexpression of CD133 protein in these lines. β-actin was used as loading control. **v**. CD133 knockdown in GBML8 led to reduced transduction with CD133-LV (MOI = 5). Conversely, CD133 overexpression in GBML3 increased the rate of transduction by CD133-LV (MOI = 5).
(PDF)

S6 Fig ***In vivo*** **validation of CD133-LV selectivity in human GBM xenografts in the mouse brain.** Intracranial xenograft tumors were generated using injection of (**A**) GBML8 cells, or (**B**) U87-MG cells (5×10^5^ cells/animal). Tumor formation (arrowhead) was confirmed with small animal MRI 2 months or 2 weeks after injection respectively. **A**. CD133-LV - transduced GBML8 xenograft cells expressing TagBFP (red) show cell surface immunoreactivity for CD133 (green). Inset shows a magnified confocal image of one of the TagBFP+ cells within the tumor. **B**. High-titer stocks of mCherry-expressing CD133-LV or VSVG-LV were injected into U87-MG intracranial tumor xenografts. CD133-LV - transduced cells expressing mCherry (red) can be observed in low abundance (arrowheads), consistent with the low CD133+ content of U87-MG cells (0.1±0.0%). In contrast, VSVG-LV produced widespread transduction (n = 3 animals per condition). Nuclei were counterstained with DAPI.
(PDF)

S7 Fig **CD133-LV selectively transduces CD133+ human melanoma cells.** **A**. Flow cytometric analysis of human melanoma cultures transduced with either CD133-LV or VSVG-LV expressing TagBFP indicates enrichment of CD133+ cells among the transduced TagBFP+ population. **B**. VSVG-LV shows significantly higher rates of transduction of human melanoma cells compared to CD133-LV, similar to its effects on human GBM cells.
(PDF)
