# Supplementary figures and images for: Selective Lentiviral Gene Delivery to CD133-Expressing Human Glioblastoma Stem Cells
Source: PLoS One. 2014 Dec 26;9(12):e116114. doi: 10.1371/journal.pone.0116114 (PMC4277468; doi:10.1371/journal.pone.0116114)

**A** Phase contrast

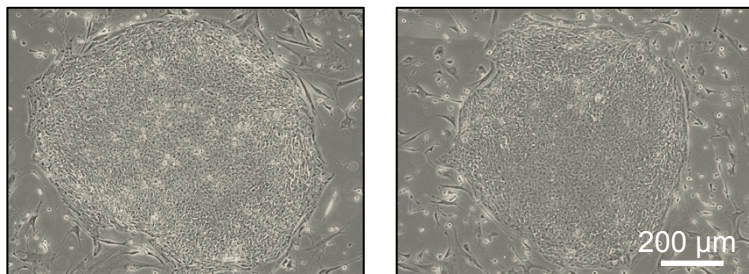

**B** DAPI merge

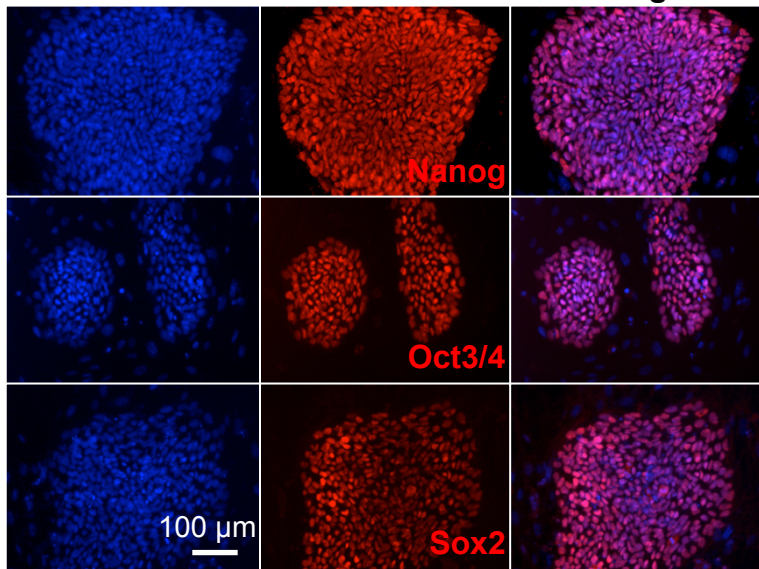

**C** hESCs rosettes neurons

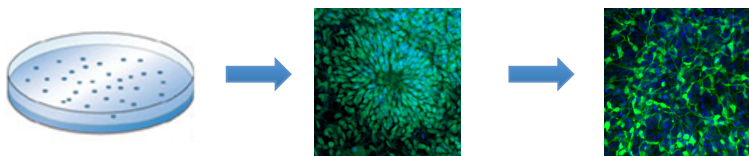

**D** DAPI ZO1 PLZF merge

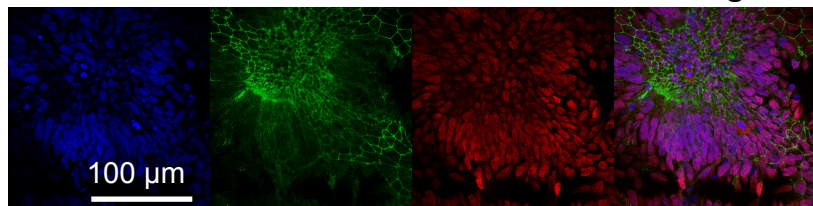

Supplement: S1 Fig — Neuronal differentiation of hESCs. A. Phase contrast microscopic images of hESC colonies grown on a layer of MEF feeders. B. Immunofluorescent microscopy shows that undifferentiated hESCs express the pluripotency-associated transcription factors Nanog, Oct3/4 and Sox2. C. The protocol utilized for generation of human neurons involves conversion of hESCs to rosette-type neural precursor cells, followed by neuronal differentiation in the presence of BDNF and AA. D. Rosettes show cell membrane immunoreactivity for the tight junction protein ZO1 (green) and transcription factor PLZF (red). (PDF) [file pone.0116114.s001.pdf]

**A**

|        | Culturing period | CD133 content (%) |
|--------|------------------|-------------------|
| GBML3  | >2 years         | $1.7 \pm 0.1$     |
| GBML8  | >2 years         | $46.5 \pm 5.7$    |
| GBML20 | >1.5 years       | $68.4 \pm 9.8$    |
| GBML27 | >1 year          | $1.4 \pm 0.4$     |

**B**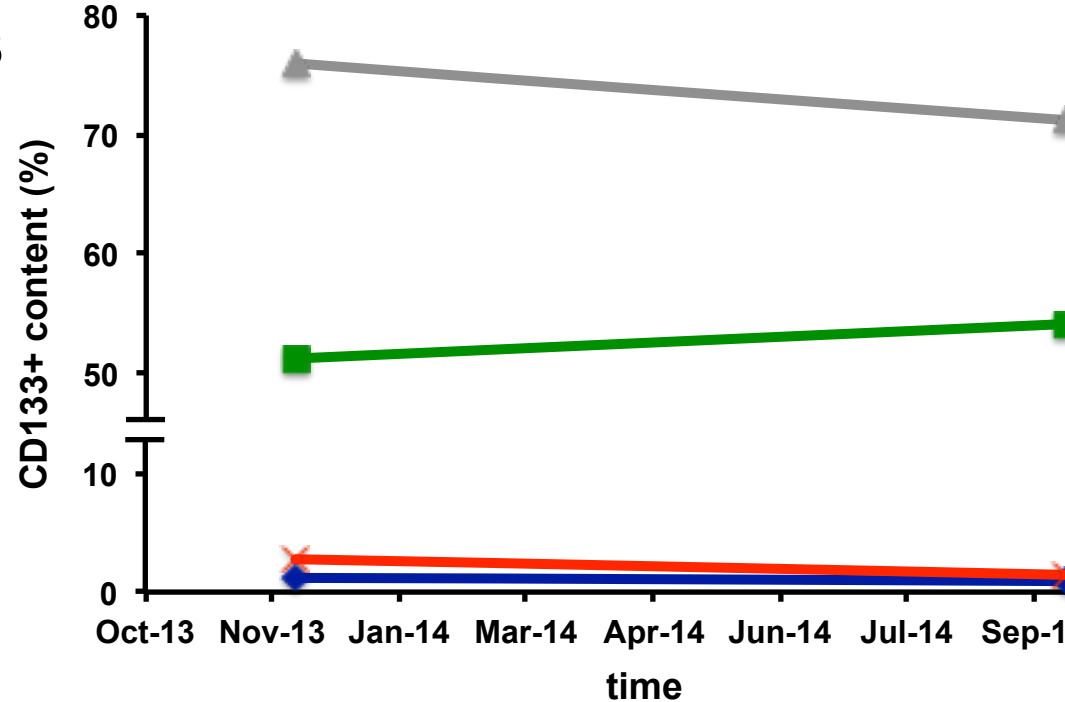**C**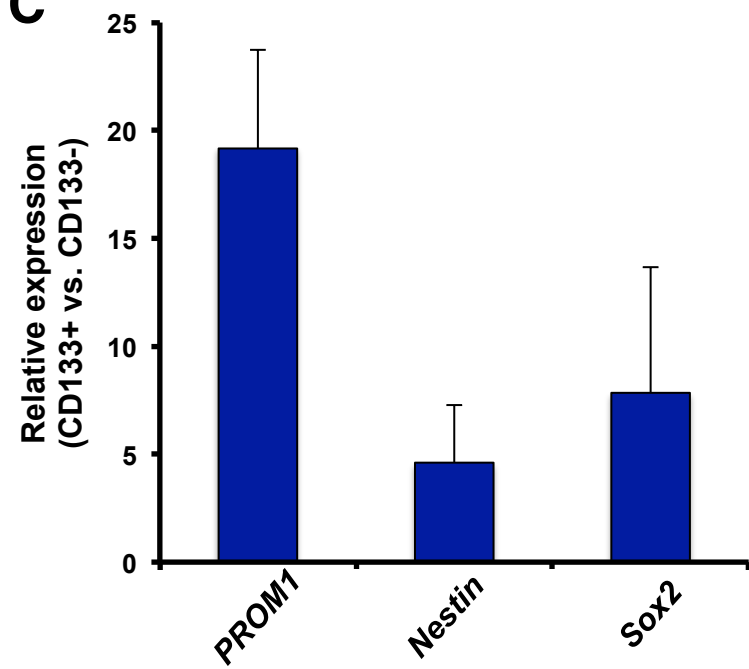**D**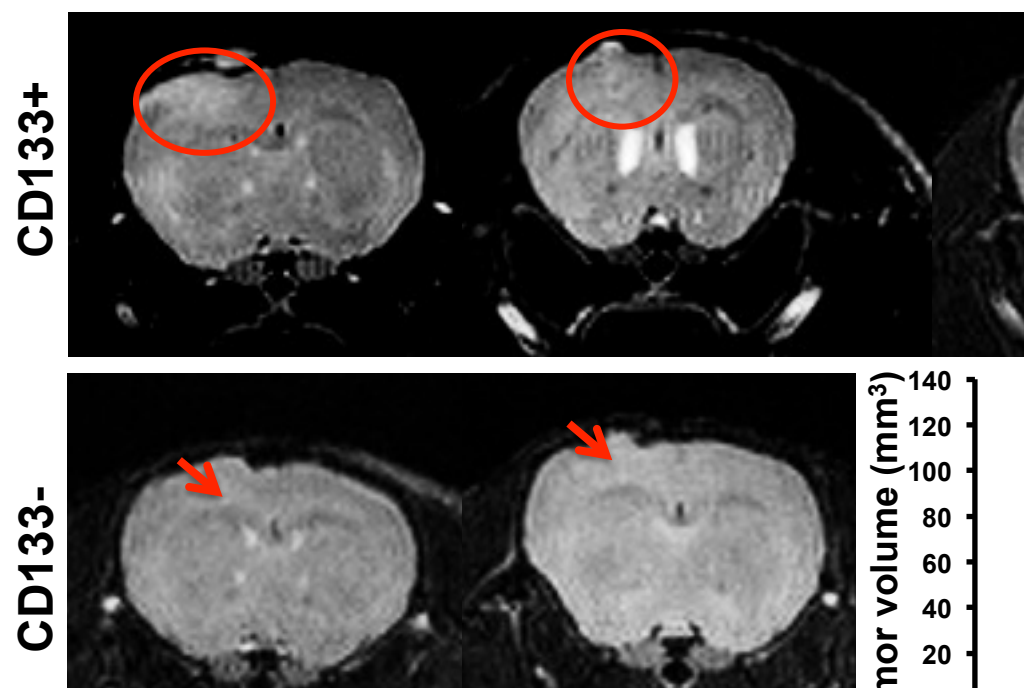

Supplement: S4 Fig — Primary GBM cultures from human biospecimens and characterization of CD133-expressing GSCs. A. Table summary of 4 different primary GBM cultures used in this study. B. Stability of CD133% content of primary GBM cultures over a period of 11 months. C. CD133+ cells from GBML3 were FACS-isolated and subjected to qRT-PCR analysis for GSC-associated transcripts (PROM1, Nestin and Sox2). D. FACS-isolated CD133+ and CD133- cells from GBML8 were injected (15,000 cells/animal) into NOD.SCID mice brains (n = 3 and n = 2 respectively). MRI analysis 4 months after injection revealed that CD133+ cells gave rise to larger tumors (circles) compared to CD133- cells (arrows), suggesting enhanced tumorigenic potential. (PDF) [file pone.0116114.s004.pdf]

**A**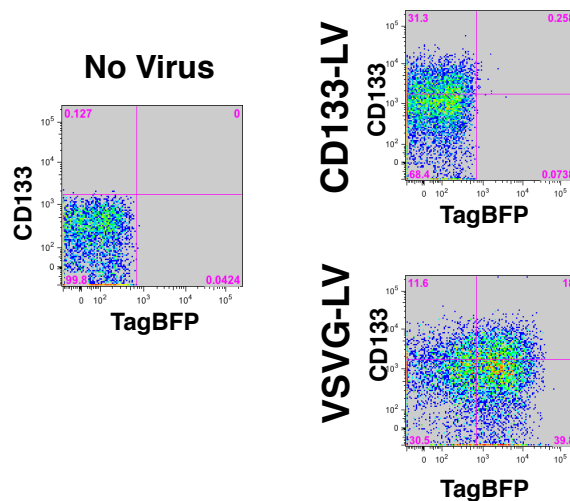**B**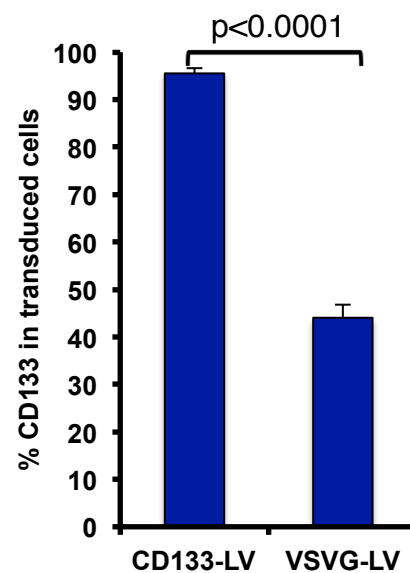**C**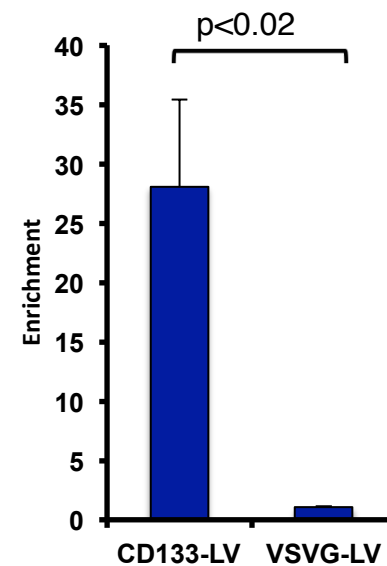**D****Flow cytometry****CD133 Knockdown**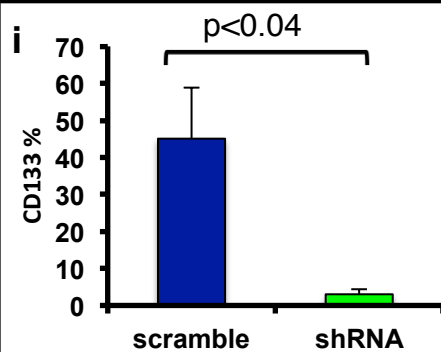**qRT-PCR**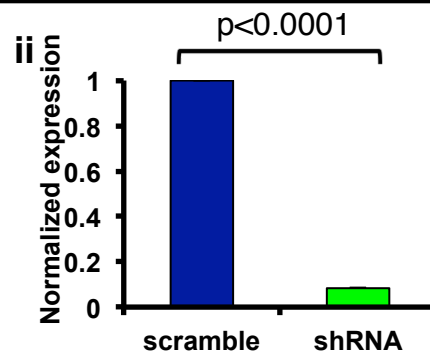**Western blot**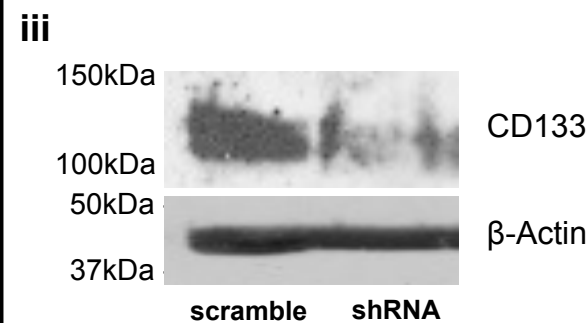**CD133-LV tr**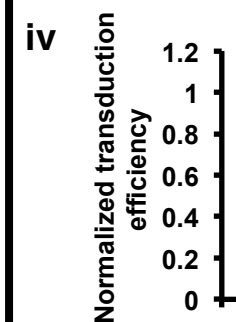**E****CD133 Overexpression**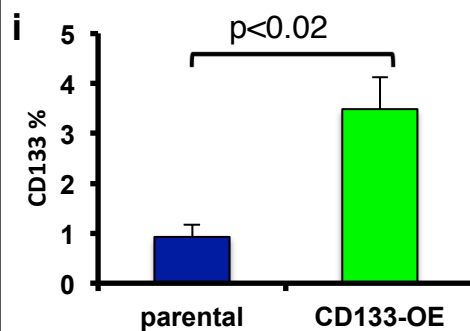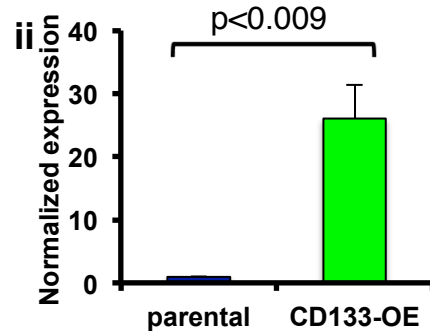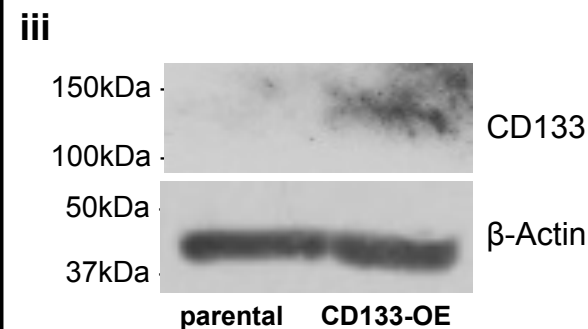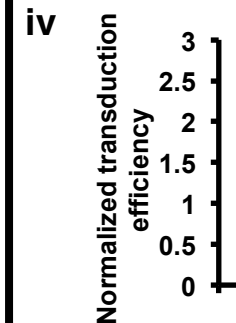

Supplement: S5 Fig — In vitro validation of CD133-LV selectivity in multiple primary GBM cultures. A. Flow cytometry analysis with GBML8 (CD133 content 45.6±5.7%) shows that CD133-LV - transduced cells (TagBFP+) are also positive for CD133 (right top). In contrast, CD133+ cells are not enriched in the cohort transduced with VSVG-LV (right bottom). Untransduced cells show no TagBFP expression as expected (left panel) B. i. Percent CD133 positivity within the transduced fraction of GBML8 with CD133-LV and VSVG-LV (MOI = 1). C. Population statistics for enrichment of CD133+ cells within the populations transduced by either CD133-LV or VSVG-LV (MOI = 1). D,E. Primary GBM lines were modified with lentiviral constructs to either knock down (D) or overexpress (E) CD133. GBML8 (CD133 content 45.6±5.7%) was used for shRNA-mediated knockdown of CD133. GBML3 (CD133 content 1.7±0.1%) was used for CD133 overexpression after transduction with lentiviral vector CD133-OE. i. Flow cytometric analysis showing the CD133+ content of primary lines expressing shRNA against CD133 or overexpressing CD133. ii. qRT-PCR analysis confirmed knockdown and overexpression of PROM1 mRNA. iii. Western Blotting validated knockdown and overexpression of CD133 protein in these lines. β-actin was used as loading control. v. CD133 knockdown in GBML8 led to reduced transduction with CD133-LV (MOI = 5). Conversely, CD133 overexpression in GBML3 increased the rate of transduction by CD133-LV (MOI = 5). (PDF) [file pone.0116114.s005.pdf]

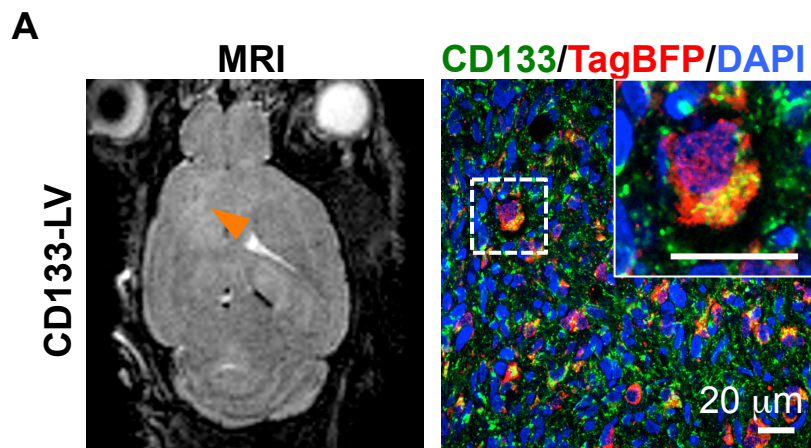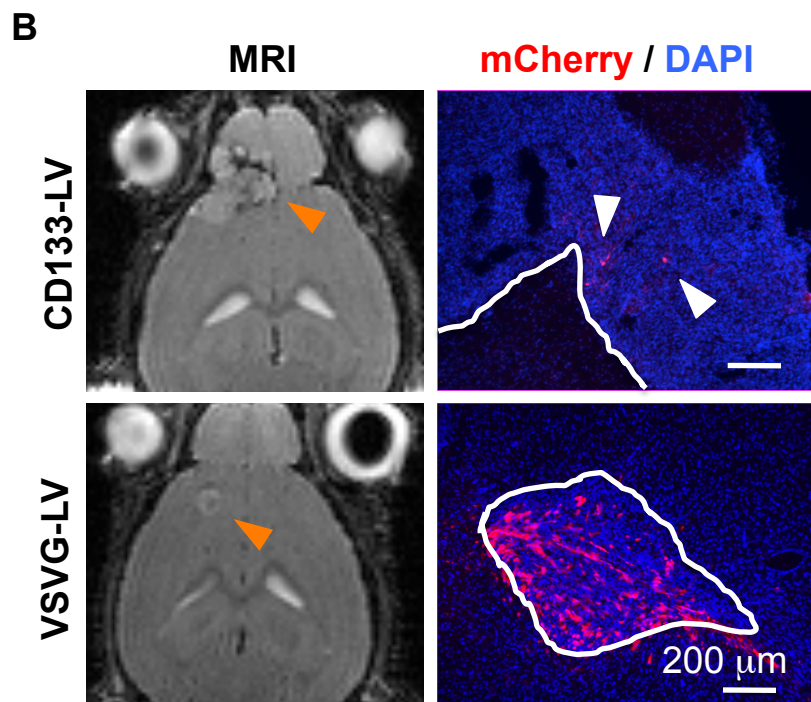

Supplement: S6 Fig — In vivo validation of CD133-LV selectivity in human GBM xenografts in the mouse brain. Intracranial xenograft tumors were generated using injection of (A) GBML8 cells, or (B) U87-MG cells (5×10^5^ cells/animal). Tumor formation (arrowhead) was confirmed with small animal MRI 2 months or 2 weeks after injection respectively. A. CD133-LV - transduced GBML8 xenograft cells expressing TagBFP (red) show cell surface immunoreactivity for CD133 (green). Inset shows a magnified confocal image of one of the TagBFP+ cells within the tumor. B. High-titer stocks of mCherry-expressing CD133-LV or VSVG-LV were injected into U87-MG intracranial tumor xenografts. CD133-LV - transduced cells expressing mCherry (red) can be observed in low abundance (arrowheads), consistent with the low CD133+ content of U87-MG cells (0.1±0.0%). In contrast, VSVG-LV produced widespread transduction (n = 3 animals per condition). Nuclei were counterstained with DAPI. (PDF) [file pone.0116114.s006.pdf]

**A**

No antibody  
No virus

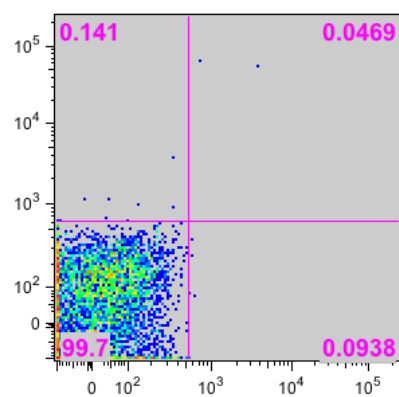

MOI

0

1

5

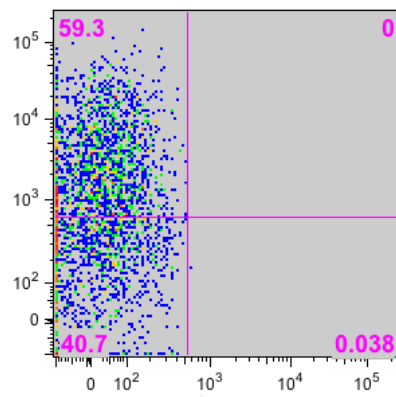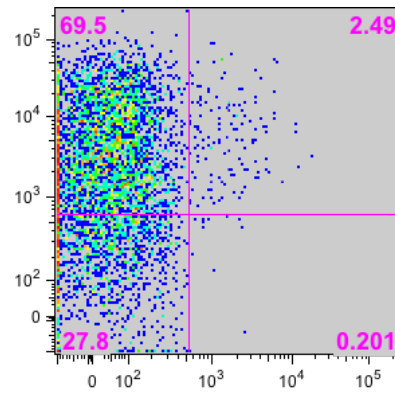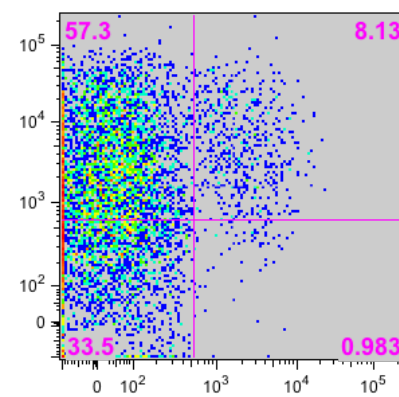

CD133-LV

**B**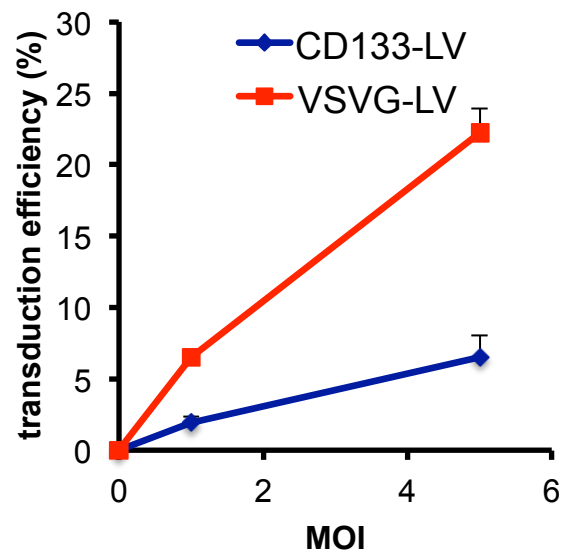

CD133

TagBFP

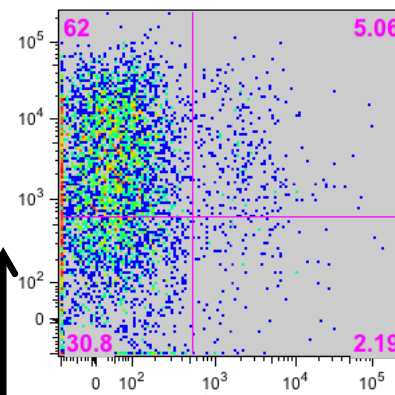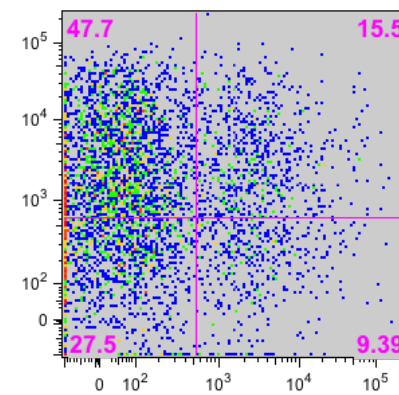

VSVG-LV

Supplement: S7 Fig — CD133-LV selectively transduces CD133+ human melanoma cells. A. Flow cytometric analysis of human melanoma cultures transduced with either CD133-LV or VSVG-LV expressing TagBFP indicates enrichment of CD133+ cells among the transduced TagBFP+ population. B. VSVG-LV shows significantly higher rates of transduction of human melanoma cells compared to CD133-LV, similar to its effects on human GBM cells. (PDF) [file pone.0116114.s007.pdf]
