# Supplementary material for: Selective Lentiviral Gene Delivery to CD133-Expressing Human Glioblastoma Stem Cells
Source: PLoS One. 2014 Dec 26;9(12):e116114. doi: 10.1371/journal.pone.0116114 (PMC4277468; doi:10.1371/journal.pone.0116114)
Supplement: S2 Fig — Lentiviral genomes. A. Architecture of the viral genomes used in this study (LTR: long terminal repeat, ψ: psi sequence, SD: splice donor, RRE: Rev response element, cPPT: central polypurine tract, WPRE: woodchuck hepatitis virus posttranscriptional response element, SIN3′-LTR: self-inactivating 3′-long terminal repeat). B. List of promoters and transgenes used in lentiviral vectors (SFFV: spleen focus-forming virus, CMV: cytomegalovirus, EF1α: eukaryotic elongation translation factor 1 alpha). (PDF) [file pone.0116114.s002.pdf]

A

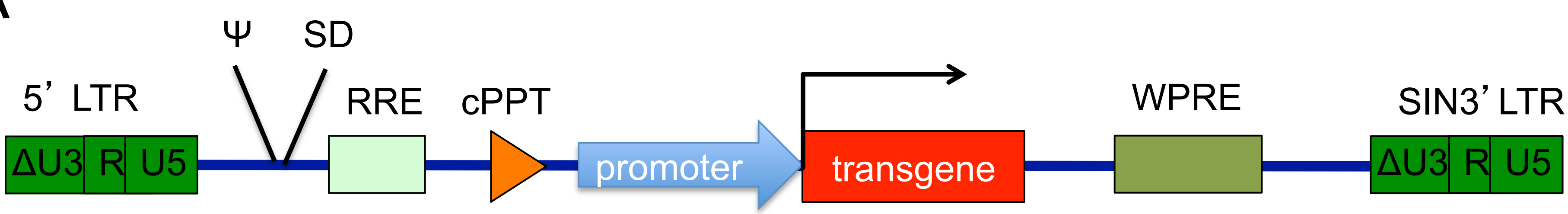

B

	Promoter	Transgene
TagBFP-expressing CD133-LV and VSVG-LV	SFFV	TagBFP
mCherry-expressing CD133-LV and VSVG-LV	CMV	mCherry
CD133-OE	EF1α	CD133 cDNA
shRNA-LV	U6	Anti-CD133 shRNA or scramble shRNA
