# Supplementary material for: Selective Lentiviral Gene Delivery to CD133-Expressing Human Glioblastoma Stem Cells
Source: PLoS One. 2014 Dec 26;9(12):e116114. doi: 10.1371/journal.pone.0116114 (PMC4277468; doi:10.1371/journal.pone.0116114)
Supplement: S3 Fig — Envelope and packaging plasmids for the production of lentiviral vectors. A. Envelope plasmids used for generation of CD133-LV. B. Envelope plasmid for VSVG-LV. C. Packaging plasmids (PCMV: CMV promoter, RRE: Rev response element, PRSV: Rous sarcoma virus promoter, pA: polyadenylation site). (PDF) [file pone.0116114.s003.pdf]

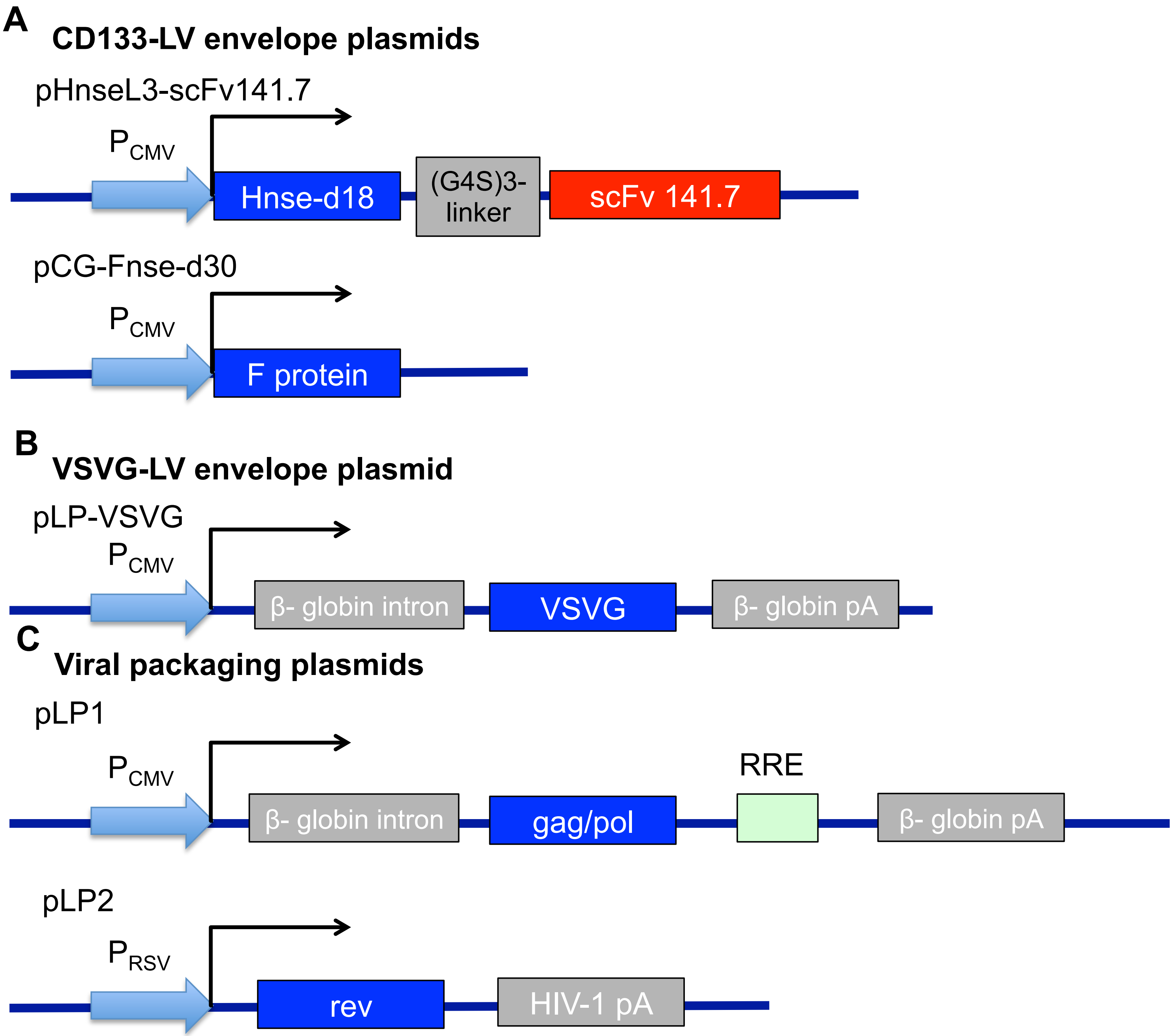
A
CD133-LV envelope plasmids
pHnseL3-scFv141.7
P CMV
Hnse-d18
(G4S)3-linker
scFv 141.7
pCG-Fnse-d30
P CMV
F protein
B
VSVG-LV envelope plasmid
pLP-VSVG
P CMV
β- globin intron
VSVG
β- globin pA
C
Viral packaging plasmids
pLP1
P CMV
β- globin intron
gag/pol
RRE
β- globin pA
pLP2
P RSV
rev
HIV-1 pA
